# A History of Alcohol Dependence Increases the Incidence and Severity of Postoperative Cognitive Dysfunction in Cardiac Surgical Patients

**DOI:** 10.3390/ijerph6112725

**Published:** 2009-10-27

**Authors:** Judith A. Hudetz, Kathleen M. Patterson, Alison J. Byrne, Zafar Iqbal, Sweeta D. Gandhi, David C. Warltier, Paul S. Pagel

**Affiliations:** 1 Department of Anesthesiology, Medical College of Wisconsin, Milwaukee, WI 53226, USA; E-Mails: ziqbal@mcw.edu (Z.I.); sdgandhi@mcw.edu (S.D.G.); dwarltier@mcw.edu (D.C.W.); pspagel@mcw.edu (P.S.P); 2 Clement J. Zablocki Veterans Administration Medical Center, Milwaukee, WI 53295, USA; E-Mails: kathleen.patterson@va.gov (K.M.P.); alison.byrne2@va.gov (A.J.B.); 3 Departments of Psychiatry, Behavioral Medicine, and Neurology Medical College of Wisconsin, Milwaukee, WI 53226, USA

**Keywords:** alcohol dependence, cardiac surgery, cardiopulmonary bypass, neurocognitive function

## Abstract

Postoperative cognitive dysfunction (POCD) commonly occurs after cardiac surgery. We tested the hypothesis that a history of alcohol dependence is associated with an increased incidence and severity of POCD in male patients undergoing cardiac surgery using cardiopulmonary bypass. Recent verbal and nonverbal memory and executive functions were assessed before and one week after surgery in patients with or without a history of alcohol dependence. Cognitive function was significantly reduced after cardiac surgery in patients with versus without a history of alcohol dependence. The results suggest that a history of alcohol dependence increases the incidence and severity of POCD after cardiac surgery.

## 1. Introduction

Postoperative cognitive dysfunction (POCD) occurs in as many as three quarters of patients after cardiac surgery and may persist in one third of these patients six months after discharge [1]. POCD is characterized by a decline in cognitive function such as memory, the ability to concentrate, and information processing. Patients with POCD often experience delayed transfer from the intensive care unit after surgery, prolonged hospitalization, and a longer recovery before returning to work [2,3]. These patients may also experience impaired self-care, increased dependency, greater attrition from rehabilitation, and higher rates of hospital readmission. The mechanisms responsible for POCD after surgery are multifactorial, and include advanced age, low educational level, preexisting cognitive impairment, and severity of coexisting illness [4–6]. Alcohol dependence is associated with frontal lobe atrophy, hypometabolism, biochemical (metabolite, and neurotransmitter/modulator), and microstructural abnormalities [7–13]. These alterations contribute to impaired memory and executive functioning that may be detectable by neuropsychological testing [10,14,15]. Alcohol dependence may also produce direct neurotoxic effects and lead to the development of alcohol-related dementia [16,17]. Self-reported alcohol dependence has been identified as a risk factor for postoperative delirium [18] and POCD after noncardiac surgery [5], but whether a history of alcohol dependence is a major risk for POCD in cardiac surgical patients has not been investigated. The current investigation tested the hypothesis that self-acknowledged alcohol dependence increases the incidence and severity of POCD in older patients undergoing cardiac surgery using cardiopulmonary bypass.

## 2. Methods

The study was approved by the Institutional Review Board of the Clement J. Zablocki VA Medical Center, Milwaukee, WI, USA. All subjects provided written informed consent.

### 2.1. Participants

One hundred and thirty eight male patients (US Armed Services Veterans) were screened; 112 consented, nine withdrew consent, and 103 completed the study. Twenty-six of 77 patients scheduled for cardiac surgery reported a history of alcohol dependence. An additional group of 26 patients without a history of alcohol dependence were included as nonsurgical controls. A subset of subjects included in the current study was recruited for other investigations [19,20]. The inclusion of the nonsurgical control group was important to account for practice effects as a result of repeated cognitive testing [21]. Prospective patients were included if they were at least 55 years of age, provided written informed consent, and were scheduled for elective coronary artery bypass graft surgery with or without valve repair or replacement using cardiopulmonary bypass. Prospective participants were excluded if they had a history of cerebrovascular accident within three years of randomization, permanent ventricular pacing, or previously documented cognitive deficits. Patients with hepatic impairment (aspartate aminotransferase or alanine aminotransferase more than twice the upper normal limit) and chronic renal insufficiency (creatinine > 2 mg/dL) were also excluded from participation.

A history of self-reported admission to a detoxification treatment facility related to alcohol consumption was used to define alcohol dependence. All participants with such a history met the criteria for alcohol dependence, but not for alcohol-induced persisting amnesic disorder or dementia using Diagnostic and Statistical Manual of Mental Disorders—Fourth Edition (DSM-IV) definitions. All participants included in the alcohol dependence group were in full remission and reported abstinence from alcohol for at least five weeks before inclusion in the study. Based on chart review, patients with a negative history of alcohol treatment did not have a diagnosis of alcohol use disorder. Nonsurgical patients without a history of alcohol dependence were recruited from various outpatient clinics by the recommendation of the head nurses who prescreened the patients for the study criteria. All participants were screened for physical health and psychological history and were excluded if there was evidence of organic brain syndrome or preexisting dementia.

### 2.2. Procedure

Neurologic status, delirium, and neurocognitive functions were assessed within one week before surgery. A neurological examination was performed to exclude patients with potential cerebrovascular anomalies. Delirium was monitored and reassessed up to a maximum of five days after surgery by three independent psychologists. If the first two psychologists disagreed, then the third psychologist’s rating determined whether or not the patient suffered from postoperative delirium. If a patient received different delirium scores on any postoperative day from the separate observers, these scores were averaged. The first two psychologists agreed with the diagnosis of delirium 100% of the time; therefore, the interrater consistency between them was 100%. Recent verbal and nonverbal memory and executive functions were assessed as indices of neurocognitive function.

Anesthetic management and cardiopulmonary bypass were standardized as follows. Patients received midazolam (1–2 mg) for premedication, and fentanyl and etomidate for anesthetic induction. Isoflurane (0.4–1.6%) and fentanyl were used for maintenance of anesthesia. All patients underwent a standard median sternotomy for cardiac surgery. Myocardial protection was produced using antegrade and retrograde cold blood cardioplegia provided at regular intervals (15 min), topical hypothermia (slushed 0.9% saline), and systemic hypothermia (30 to 32 degrees centigrade).

A dose of continuous warm blood cardioplegia was administered during rewarming before removal of the aortic cross clamp. Cardiopulmonary bypass flows were 2.4 to 2.5 L/min/m^2^. Mean arterial pressure was maintained between 55 and 70 mmHg during CPB. Heparin (400 U/kg) was used for systemic anticoagulation to maintain the activated clotting time (ACT) > 500 sec. Neurological status and neurocognitive functions were reassessed one week after surgery or at hospital discharge, whichever occurred first or one week apart for patients who did not undergo surgery. The psychologists performing the pre- and postoperative neurocognitive assessments and physicians performing the neurological examinations were blinded to each participant’s history of alcohol use.

### 2.3. Delirium, Neurocognitive, Depression, and Neurological Testing

Delirium was assessed with the Intensive Care Delirium Screening Checklist [22]. This is an 8-item (altered level of consciousness, inattention, disorientation, hallucination-delusion-psychosis, psychomotor agitation or retardation, inappropriate speech or mood, sleep/wake cycle disturbance, symptom fluctuation) checklist based on DSM-IV criteria and features of delirium. Raters completed the checklist based on data from the previous 24 hrs. Routinely collected data (such as orientation) were combined with short observations of obvious manifestations of described features. The eight items are scored 1 (present) or 0 (absent) for a total of 8 points. A score of 4 or greater is considered a positive screen for delirium [22].

The neurocognitive test battery was comprised of standard clinical measures that were appropriate for use with subjects in the age group studied, with minimal sensory or motor demands. The tests covered three cognitive domains: verbal recent memory, nonverbal recent memory, and executive functions. Total test administration time was less than one hour. When available, alternate forms (A and B) were used to reduce practice effects between sessions. The order of presentation was counterbalanced with half of the subjects receiving form A at the first session and the other half receiving form B.

To test verbal recent memory, Story Memory and Word List Memory subtests from the Repeatable Battery for the Assessment of Neuropsychological Status [23] were used. Story Memory measures the ability to learn and recall a narrative story in two trials (maximum score 20) and delayed free recall (maximum score 10). Word List Memory assesses the ability to learn and remember a list of 10 unrelated words across four sequential learning trials (maximum score 40) and delayed free recall (maximum score 10).

To test nonverbal recent memory, the Brief Visual Memory Test-Revised [24] was used. Participants were shown a card with six simple geometric designs (2 points per design) for ten seconds on three sequential trials. When the card was removed, the examinee drew the designs in the locations remembered (maximum score 36). A free recall trial was administered twenty minutes later (maximum score 12).

To test executive functions, Semantic Fluency [23], Phonemic Fluency [25], and Digit Span (backward) [26] were used. Semantic and Phonemic Fluency are subtests of the Delis-Kaplan Executive Function System [27] that examine executive functions related to language. Semantic Fluency measures executive speed of word generation using semantic cues such as identifying all the “fruits and vegetables” (form A) or “animals in the zoo” (form B) that the patient is able to produce in 1 min. The obtained score is the number of appropriate words generated within the time interval. Phonemic Fluency measures executive speed of word generation using phonetic cues such as identifying all the words that start with the letter “S” (form A) or “P” (form B) that the patient is able to produce in 1 min. The score is the number of appropriate words identified within the time interval. Digit Span is a subtest of the Wechsler Adult Intelligence Scale-Third Edition [26] and measures attention span, concentration, and the Backward Digit Span executive functions.

Depression was assessed with the Geriatric Depression Scale 15-item version [28]. The obtained score was the number of symptoms endorsed.

Neurological examination focused on detection of significant visual and auditory impairment, level of consciousness, cranial nerve function, motor strength, cerebellar ataxia, intention tremor, sensation, frontal lobe release signs, deep tendon and plantar reflexes, and gait ataxia. An additional total score was calculated using the Hachinski Ischemia Scale [29]. A score greater than 4 is diagnostic of vascular dementia.

### 2.4. Statistical Analysis

Power analysis was conducted based on a pilot study of 28 subjects. POCD based on composite z-scores was observed in 57% and 14% of patients with and without a history of alcohol dependence. Assuming 80% power and a 2-sided alpha of 0.05, power analysis yielded *N* = 23 as the estimated number of subjects per group to detect a difference in cognitive function between those with and without a history of alcohol dependence. An attrition rate of 10% was estimated to account for those whose second neurocognitive testing could not be performed due to dropout; thus, we recruited 1.1 times 23 yielding a total of at least 26 subjects per group.

To test for between-group differences in the demographic and medical data, Chi-square or Fisher’s exact tests were used for proportions and the Student’s t-test was used for continuous outcomes.

To determine the presence of POCD, we followed Moller’s method [4]. The z-scores express by how many standard deviations the surgical patient’s performance deviates from the expected in the control patients. The z-scores were calculated as differences in all test parameters between the pre- and postoperative test sessions for the individual patients with appropriate signs (plus or minus). After subtracting the mean change between sessions in the nonsurgical control group, which represents the practice effect, the result was then divided by the standard deviation for this change in the nonsurgical control group. The use of a suitable normative population is essential to allow correction for practice effects and variability between sessions [21]. To obtain composite z-scores, we averaged all practice-effect controlled z-scores of all tasks that comprised the test battery for the individual surgical patients. The composite z-score represents a general or global measure of the domains of functioning assessed and represents their operationalized definition of POCD. Patients were defined as having cognitive dysfunction when their composite z-score was less than −1.96 (2 standard deviations). This definition took into account general postoperative deterioration in performance across all tests. Patients with or without a history of alcohol dependence who met the 2 *SD* criterion were compared using the Chi squared test. Cognitive performance between groups was also compared using the Mann-Whitney *U*-test based on composite z-scores. In addition, we conducted repeated measures of analysis of variance (Table 3) with covariates of smoking status, age, and sleep disorder to determine the significance of group change on the individual measures. None of the covariates revealed significance; therefore we dropped them from the model. To account for multiple comparisons we used the Bonferroni correction. To reveal which neurocognitive tests were indicative of most profound impairment in patients with a history of alcohol dependence, effect sizes (Cohen’s *d*) were calculated. Because depression scores are not normally distributed, these values were expressed as median and confidence intervals (CI), and the Mann-Whitney *U*-test was used for between group comparisons. Multiple linear regression analysis was performed to examine the possible effects of demographic, medical, and perioperative conditions on the cognitive test results (composite z-scores). Significant (*p* < 0.05) demographic variables, perioperative predictors that showed *p* = 0.1 or less significance in a univariate regression model, and age were entered into the final multivariate model.

All comparisons were made using 2-sided tests at α < 0.05. All errors are reported as *SD*. Statistical calculations were performed using NCSS 2001 (NCSS, Kaysville, UT) and STATA/IC 10.0 (StataCorp LP, College Station, TX) software.

## 3. Results

A total of 77 patients were enrolled in the surgical groups (51 without and 26 with a history of alcohol dependence). An additional group of 26 patients was included that did not undergo cardiac surgery. Demographics were similar between groups (Table 1). Patients with a history of alcohol dependence had a significantly (*p* = 0.03) greater incidence of tobacco abuse and were more likely (*p* = 0.03) to be affected by a sleep disturbance than their counterparts. Surgical, anesthesia, and postoperative care variables were similar between patients with and without a history of alcohol dependence with the single exception of the duration of intensive care unit stay (*p* = 0.01) (Table 2).

There was no significant (*p* = 0.07) difference in the incidence of postoperative delirium between patients with versus without a history of alcohol dependence (Table 2). Postoperative delirium occurred within 72 hrs after surgery in 80% of patients. All cases of postoperative delirium were observed within five days of surgery. No patients displayed symptoms of postoperative delirium during neurocognitive testing one week after surgery. Baseline neurocognitive and depression scores were similar between groups (Table 3). Cognitive performance after surgery decreased by at least 2 *SD*s (composite z-score of 1.96) in nine patients (18%) without and 14 patients (54%) with a history of alcohol dependence (*p* < 0.001 versus nonsurgical patients; data not shown). The nonsurgical patients’ data are reported elsewhere [19]. Cognitive performance was significantly (*p* < 0.001) different between patients with versus without a history of alcohol dependence based on composite z-scores. Effect sizes (Cohen’s *d*) for the neurocognitive measures revealed large (>0.8) effect in 1 test, medium (0.5–0.8) effect in 6 tests, and small (0.2–0.4) effect in 2 tests in the alcohol dependence group between the two testing periods (Table 3). In contrast, depression scores were unchanged after surgery in both groups (Table 3). Variables that revealed *p* = 0.1 or less significance between the predictor and composite z-scores in the univariate regression model from Table 4 (history of alcohol dependence, incidence of postoperative delirium, and intensive care unit stay) and significant demographic variables from Table 1 (cigarette smokers and people with sleep disorders) were entered into the multiple regression model. Multiple regression analysis revealed that the history of alcohol dependence and incidence of postoperative delirium significantly (*p* < 0.001) predicted the change in composite z-scores. After age was included in the model, history of alcohol dependence and postoperative delirium remained significant (*p* < 0.001) predictors of the change in composite z-scores.

## 4. Discussion

The results of the current investigation demonstrate that a history of alcohol dependence requiring hospitalization for detoxification is associated with an increased incidence and severity of POCD one week after cardiac surgery using cardiopulmonary bypass. These findings were observed despite similar preoperative cognitive function in patients with versus without a history of alcohol dependence, suggesting that preexisting alcohol-induced cognitive abnormalities did not contribute to the current results. POCD has been identified as an important syndrome that affects many cardiac surgical patients [1,30] and has potentially profound consequences for subsequent quality of life [31]. Within this context, the current data indicate that a history of alcohol dependence is a major preoperative risk factor for POCD in the setting of cardiac surgery that should be anticipated in this patient population. These results confirm and extend those of previous studies demonstrating that alcohol dependence increases the risk of postoperative delirium and POCD in patients undergoing noncardiac surgery [18,19].

The neurocognitive tests in the current investigation were designed to measure performance in recent verbal and nonverbal memory and executive functions because previous studies suggested that cognitive impairments may develop in these domains after surgery [10,32–36]. The current investigation was specifically conducted in older patients (age ≥ 55 years) who are known to be at substantially greater risk for POCD [1,37–40]. Advanced age is associated with reduced brain weight and volume, loss of cellular bodies and myelinated fibers in several brain regions (e.g., hippocampus), synaptic density, and DNA repair capability [41–44]. Patients with a history of alcohol dependence may also demonstrate preexisting cognitive impairment [10,32,33,36,39,45–52]. For example, impaired visuospatial recent memory and the ability to learn new verbal material were previously described in abstinent alcoholics [32,33,39]. Similarly, executive dysfunction may be another characteristic sign of chronic alcohol dependence [10,36]. Dysfunction in recent memory may result from impairments in hippocampi, entorhinal cortices, thalami, and the basal forebrain [53]. Executive functions depend on intact prefrontal cortices, including dorsolateral white matter tracts [54]. Thus, it appears that a history of alcohol dependence may impair some or all these structures simultaneously [55]. Differences in the degree of impairments on various cognitive tests as observed in different individuals may reflect site-specific variability in cognitive reserve [55]. It is interesting that we did not find baseline differences in cognitive performance between patients with versus without a history of alcohol dependence. The lack of cognitive reserve seems to be a potential explanation for the greater frequency of POCD in patients with a history of alcohol dependence, particularly given the equivalence of groups on education and baseline cognitive testing. Specifically, it is possible that the level of alcohol consumption in the alcohol dependent group served as an addition stress/burden/injury to their brain neurobiology in the context of the stress exerted by the rigors of cardiac surgery. Consequently, the “cognitive reserve” of many of these individuals may have been unable to compensate. Executive function deficits, in particular, have been shown to predict difficulty with postoperative rehabilitation [56], and these cognitive functions were substantially affected in postoperative cardiac surgical patients with a history of alcohol dependence.

The participants with a history of alcohol dependence in the current investigation reported that they had not consumed alcohol for at least five weeks before initial cognitive testing was conducted. Nevertheless, long-term deficits in the learning of novel associations may be present even after prolonged periods of abstinence [40]. Previous neuropsychological and imaging studies demonstrated dysfunction in mediofrontal and in the left dorsolateral prefrontal cortex in patients with chronic alcohol dependence in the absence of explicit neurological symptoms [9,10,57]. Similarly, neurocognitive functions were impaired in patients with a history of alcohol dependence after cardiac surgery despite an apparent absence of preoperative neurological abnormalities.

Postoperative neurologic deterioration is common in cardiac surgical patients, especially when cardiopulmonary bypass is used. Reduced perfusion pressure during cardiopulmonary bypass places patients at risk of cerebral ischemia. Embolization of air or particulate matter during aortic cannulation or weaning from cardiopulmonary bypass may produce focal neurological damage and neuropsychiatric complications. [58] Cardiopulmonary bypass also causes a systemic inflammatory response, which may contribute to the development of neurologic injury [59,60]. This systemic inflammation may be mediated by surgical trauma, blood contact with the extracorporeal bypass circuit, and lung reperfusion injury after discontinuation of cardiopulmonary bypass. The mechanism(s) responsible for POCD remain unclear, but a combination of effects from anesthetics, analgesics, sleep deprivation, recovery after major surgery, and factors associated with the hospital environment are most likely important contributing factors. The presence of preexisting diseases also contributes to the development of POCD. As such, the observation that patients with a history of alcohol dependence required longer length of stay in the intensive care unit may be indicative of a greater number of comorbidities. Patients with a history of alcohol dependence had a higher incidence of sleep disturbances before surgery; it is possible that this abnormality contributed to more pronounced postoperative sleep deprivation than their counterparts, thereby negatively impacting cognitive performance. In the current investigation, POCD was also associated with postoperative delirium independent of a history of alcohol dependence. Whether general anesthesia itself contributed to the development of POCD in the patients with versus without a history of alcohol dependence is unclear, but the conduct of anesthesia was standardized between groups to minimize this potential confounding factor. The current investigation did not evaluate whether differences in short-term cognitive functions between patients with or without a history of alcohol dependence persisted to become long-term changes in cognition. The results must also be qualified because female patients were not included in the current investigation.

## 5. Conclusions

In conclusion, the current results suggest that a history of alcohol dependence in patients ≥55 years of age increases the incidence and severity of POCD after cardiac surgery using cardiopulmonary bypass. Additional studies will be required to determine the duration or permanence of these cognitive changes and their potential impact on rehabilitation and quality of life in this vulnerable patient population.

## Figures and Tables

**Table 1.** Demographics.

	− Alcohol Dependence	+ Alcohol Dependence	

	*N* = 51	*N* = 26	*p* value

Age, yr	69 ± 8	65 ± 8	0.07
Height, cm	176 ± 6	176 ± 6	0.89
Weight, kg	94 ± 17	90 ± 18	0.30
Education, yr	13 ± 3	13 ± 2	0.80
Caucasian (%)	47(92)	23(88)	0.59
Married (%)	34(67)	15(58)	0.43
Smoking (%)	8(16)	10(38)	0.03
Right handedness (%)	45(88)	22(85)	0.65
Hypertension (%)	47(92)	23(88)	0.68
Hypercholesterolemia (%)	45(88)	25(96)	0.41
Angina (%)	19(37)	11(42)	0.66
Arrhythmia (%)	11(22)	5(19)	1.00
Myocardial infarction (%)	6(12)	7(27)	0.09
Peripheral vascular disease (%)	6(12)	2(8)	0.71
Diabetes (%)	32(62)	15(58)	0.68
Post-traumatic stress disorder (%)	1(2)	2(8)	0.26
Anxiety disorder (%)	4(8)	5(19)	0.16
Depression (%)	7(14)	7(27)	0.16
Congestive heart failure (%)	9(18)	2(8)	0.31
Stroke (%)	2(4)	2(8)	0.60
Chronic obstructive pulmonary disease (%	7(14)	3(12)	1.00
Sleep disorder (%)	13(25)	13(50)	0.03
Hatchinski score > = 4, baseline (%)	0(0)	0(0)	
Hatchinski score > = 4, 1 week (%)	0(0)	0(0)	
Preoperative medications			
Nitrates (%)	16(31)	11(42)	0.34
Beta blockers (%)	36(71)	20(77)	0.55
ACE inhibitors (%)	34(67)	16(61)	0.65
Calcium channel blockers (%)	16(31)	3(12)	0.09
Diuretics (%)	23(45)	8(31)	0.22
Lipid lowering agents (%)	44(86)	20(77)	0.30
NSAIDS (%)	2(4)	1(4)	1.00
Antiarrhythmics (%)	3(6)	0(0)	0.54

The *p* values were from *t*-test for continuous variables and Chi-square or Fisher’s exact test for dichotomous variables, data are expressed as number (%) or mean ± SD.

**Table 2.** Surgical, anesthesia, and postoperative care data in two patient groups.

	− Alcohol Dependence	+ Alcohol Dependence	

	*N* = 51	*N* = 26	*p* value

CABG	38(75)	19(73)	0.89
Valve	8(16)	3(12)	0.74
CABG and Valve	5(10)	4(15)	0.48
ASA physical status, frequency, III/IV	25/26	13/13	0.94
Ejection fraction, %	54 ± 11	54 ± 9	0.93
Anesthesia duration, min	453 ± 68	477 ± 71	0.15
Surgery duration, min	354 ± 64	380 ± 83	0.13
Cardiopulmonary bypass duration, min	149 ± 43	165 ± 57	0.15
Aortic crossclamp duration, min	119 ± 37	135 ± 43	0.10
Fentanyl dose, mcg	1185 ± 469	1104 ± 247	0.41
Isoflurane dose, % end-tidal concentration	0.78 ± 0.22	0.80 ± 0.21	0.64
Propofol (%)	50(98)	26(100)	0.47
Extubation, postoperative day 0 (%)	21(41)	1(42)	0.92
Extubation, postoperative day 1 (%)	29(57)	11(42)	0.22
Extubation, postoperative day 2 or more (%)	1(2)	3(12)	0.11
Intensive care unit stay, day	3 ± 1	4 ± 2	0.01
Delirium (%)	4(8)	6(23)	0.07
Reoperation (%)	2(4)	3(12)	0.33
Hospital stay, day	7 ± 3	8 ± 4	0.13
Postoperative hospital readmission (30 days) (%)	9(18)	4(15)	0.71
Morphine consumption, day of surgery, mg	15 ± 9	16 ± 8	0.90
Morphine consumption, postoperative day one, mg	16 ± 14	15 ± 13	0.70
Morphine consumption during postoperative testing (%)	27(53)	13(50)	0.87

ASA: American Society of Anesthesiologists. The p values were obtained from t-test for continuous variables and Chi-square or Fisher’s exact test for dichotomous variables.

**Table 3.** Neurocognitive and depression scores before and one week after surgery.

	**Baseline**		**One Week**			

	− Alcohol Dependence	+ Alcohol Dependence	− Alcohol Dependence	+ Alcohol Dependence	*p*	*d*

**Variables**						

*Nonverbal memory:*						

Figure Reconstruction	19.7 ± 6.8	19.8 ± 6.9	18.0 ± 5.9	13.8 ± 7.3	0.001*	0.87
Delayed Figure Reproduction	7.5 ± 2.7	7.1 ± 3.0	6.6 ± 2.9	4.8 ± 3.2	0.02	0.77

*Verbal memory:*						

Immediate Story Recall	18.0 ± 4.2	17.1 ± 4.1	18.0 ± 4.7	15.1 ± 6.3	0.13	0.49
Delayed Story Recall	9.2 ± 2.5	8.5 ± 2.9	8.9 ± 2.8	6.8 ± 3.5	0.03	0.59
Immediate Word List Recall	25.3 ± 7.1	26.0 ± 7.3	24.5 ± 6.4	20.4 ± 7.6	0.0001*	0.77
Delayed Word List Recall	5.3 ± 3.2	5.8 ± 2.7	4.6 ± 3.0	3.7 ± 2.6	0.009	0.78

*Executive functions:*						

Digit Span (backward)	8.1 ± 1.7	8.4 ± 2.1	8 ± 1.9	7.5 ± 2.5	0.02	0.43
Semantic Fluency	16.0 ± 3.3	15.6 ± 5.4	14.2 ± 3.7	11.5 ± 3.8	0.01	0.76
Phonemic Fluency	11.4 ± 4.7	12.1 ± 4.6	10.5 ± 5.2	8.7 ± 4.3	0.009	0.74

*GDS-15*	2(1–5)	3(1–4)	2(1–5)	3(1–5)	0.20	

Neurocognitive scores are expressed as mean ± SD and depression scores as median (interquartile range) in patients with and without a history of alcohol dependence *p*-values were obtained from repeated measures analysis of variance,

*: significance (*p* < 0.005) after Bonferroni correction, *d*: effect sizes (Cohen’s *d*) represent the change in measures between baseline and 1 week for the +Alcohol Dependence group.

**Table 4.** Results of univariate linear regression analyses.

**Composite z–score**	**Coefficient**	**Standard Error**	**t**	**p**	**R^2^**

History of alcohol dependence	−0.98	0.21	−4.53	0.001	0.21
Anesthesia duration, min	−0.001	0.002	−0.05	0.96	0.001
Surgery duration, min	0.00	0.001	−0.17	0.86	0.001
Cardiopulmonary bypass duration, min	−0.001	0.002	−0.07	0.95	0.001
Aortic crossclamp duration, min	0.001	0.002	0.3	0.76	0.001
Intensive care unit stay, day	−0.19	0.06	−3.21	0.002	0.12
Delirium (%)	−1.51	0.30	−5.1	0.001	0.27
Hospital stay, day	−0.03	0.04	−0.77	0.44	0.008
Age, yr	0.004	0.014	0.29	0.77	0.001
